# Exploration of Root-associated Bacteria from the Medicinal Plant *Platycodon grandiflorum*

**DOI:** 10.1264/jsme2.ME19030

**Published:** 2019-12-27

**Authors:** Cong-Min Huang, Wen-Ching Chen, Shih-Han Lin, Yu-Ning Wang, Fo-Ting Shen

**Affiliations:** 1 Department of Soil and Environmental Sciences, College of Agriculture and Natural Resources, National Chung Hsing University 40227 Taichung Taiwan, R.O.C; 2 International Bachelor Program of Agribusiness (IBPA), College of Agriculture and Natural Resources, National Chung Hsing University 40227 Taichung Taiwan, R.O.C; 3 Innovation and Development Center of Sustainable Agriculture (IDCSA), National Chung Hsing University 40227 Taichung Taiwan, R.O.C

**Keywords:** root-associated bacteria, *Platycodon grandiflorum*, medicinal plant, plant growth promotion, plant constituent-hydrolyzing activities

## Abstract

The present study was performed to investigate root-associated bacteria from *Platycodon grandiflorum*, a medicinal plant commonly grown in East Asia. Isolates were obtained from the rhizosphere or root interior with various culture media, and phylogenetic analyses were performed based on their 16S rDNA sequences. In consideration of practical applications, traits related to plant growth promotion and niche adaptation were assessed in several endophytic strains with fewer biosafety concerns. The effects of a bacterial inoculation on seedling and mature plant growth were evaluated. Seventeen genera that encompassed more than 30 bacterial lineages were successfully retrieved from the roots, the majority of which have not been reported as *P*. *grandiflorum*-associated bacteria, particularly for non-negligible *Proteobacteria*. Although nitrogen-fixing or phosphate-solubilizing and indole acetic acid-producing activities were recorded in all of the strains selected, these strains were beneficial or detrimental to plant growth as evidenced by their influence on the length of seedlings and biomass of mature plants. Among the 4 endophytic *Rhizobium* species tested in the present study, the potentially novel *Rhizobium* sp. BF-E16, which was more compatible with the non-leguminous medicinal plant *P. grandiflorum*, was identified. Other than plant growth-promoting traits, characteristics such as plant constituent-hydrolyzing activities need to be taken into consideration and their roles clarified when investigating plant growth-promoting rhizobacteria.

The rhizosphere is the interface between plant roots and soil at which a myriad of microorganisms and invertebrates interact with each other. These organisms affect not only biogeochemical cycling, but plant growth and tolerance to biotic and abiotic stresses (32). While many members of the rhizosphere microbiome are beneficial to plant growth, plant pathogenic microorganisms also colonize the rhizosphere to cause disease (29). Plant growth-promoting bacteria (PGPB) may promote plant growth directly by facilitating resource acquisition or modulating plant hormone levels. They may also decrease the inhibitory effects of various pathogenic agents on plant growth and development, particularly in suppressive soils (13, 41). Direct mechanisms by PGPB involve the fixation of nitrogen, solubilization of phosphate, sequestering of iron, and production of indole acetic acid (IAA), cytokinins, gibberellins, or 1-aminocyclopropane-1-carboxylate deaminase (14).

The microbiome within plant roots may significantly differ from that within the rhizosphere, suggesting that plants affect the microbial communities inside their roots (11, 16). Some endophytes have the ability to promote plant growth, and the mechanisms employed by bacterial endophytes are similar to those used by rhizospheric bacteria (36). However, limited information is currently available on the plant growth-promoting endophytes of medicinal plants (20).

*Platycodon grandiflorum* (Jacq.) A. DC. is a medicinal plant commonly grown in East Asia. It has a long history of use as a traditional herbal medicine or as an ingredient in health foods and vegetable dishes. A phytochemical investigation revealed that this product contains steroidal saponins, flavonoids, polyacetylenes, sterols, phenolics, and other bioactive compounds, indicating its strong potential for therapeutic and health-maintaining uses (45). However, only a few studies have examined bacteria associated with *P. grandiflorum*. Experiments were conducted to isolate endophytic bacteria from 1-, 3-, and 6-year-old roots, and the findings obtained demonstrated that the population of low G+C Gram-positive bacteria gradually increased by 60–80% from 1- to 6-year-old samples (2). Other isolates obtained from the rhizosphere or root interior of *P. grandiflorum* belonged to genera such as *Bacillus*, *Luteibacter*, *Mucilaginibacter*, and *Pseudomonas* (3, 6, 9, 26).

Many plant-associated bacteria promote plant growth or increase plant resistance. Nevertheless, they often fail to confer these beneficial effects when applied in the field, which is often due to insufficient rhizo- and/or endosphere colonization (8). A previous study demonstrated that the type and concentration of nitrogen fertilizer affected the colonization of rice plants by *Azospirillum* sp. B510 (30). A synthesis of available data suggested that motility, plant cell wall degradation ability, and reactive oxygen species (ROS) scavenging are crucial traits for successful endophytic colonization and the establishment of bacteria (28). In the present study, attempts were made to screen for bioinoculants compatible with *P*. *grandiflorum*. Root-associated bacteria from the rhizosphere or root interior were isolated and phylogenetically characterized. Traits related to plant growth promotion and niche adaptation were assessed in the selected strains, which were subsequently used as bioinoculants to evaluate their effects on plant growth.

## Materials and Methods

### Sample preparation and bacterial isolation

*P. grandiflorum* grown in pots was collected from a garden of medicinal plants located in central Taiwan. After removing loosely bound soil, 18 g of rhizospheric soil was collected, mixed with 45 g of sterile water in a flask, and shaken at 160 rpm for 20 min. Samples were serially diluted and plated on Nutrient Agar (Cat. No. 213000, Difco, Detroit, MI, USA), nitrogen-free agar (23), and tricalcium phosphate agar (containing [L^−1^ distilled water]: glucose 10 g, NH_4_NO_3_ 0.27 g, Ca_3_(PO_4_)_2_ 5 g, KCl 0.2 g, MgSO_4_·7H_2_O 0.1 g, FeSO_4_·7H_2_O 0.001 g, and MnSO_4_·4H_2_O 0.001 g. pH was adjusted to 7.0 before autoclaving and 2% of bacteriological agar powder was added), modified from that described by Subba Rao (38). Regarding the isolation of bacteria from the root interior, roots were surface sterilized with 75% of ethanol for 5 min followed by 30% of H_2_O_2_ for 10 min and then rinsed again with 75% of ethanol. Another rinse with sterile water was repeated 5 times and the final rinse water was used to assess the sterilization process. Root samples were pressed and effluents from the root interior were collected, serially diluted, and plated on the same culture media described above. After 4 d of cultivation at 30°C, isolates from the rhizosphere or root interior were obtained and transferred several times to assure purity. To examine the facultatively oligotrophic behavior of selected isolates, nutrient agar and 1,000-fold diluted nutrient agar were both used (23). Colony formation that represented bacterial growth after 2–7 d of cultivation was monitored.

### Phylogenetic analysis

Genomic DNA was isolated from all isolates using the UltraClean Microbial Genomic DNA Isolation Kit (MO BIO Laboratories, Carlsbad, CA, USA). 16S rDNA was amplified by PCR with bacterial universal primers (10), purified, and sequenced as described by Huang and Shen (23), and bacterial identification was then performed by comparing the 16S rDNA sequences of isolates and their most closely related type strains using the EzBioCloud Identify function (44). Sequences were aligned using ClustalX program version 2 (27) and distances and clustering with the neighbor-joining method were performed using the software package MEGA version 6 (39). The 16S rDNA sequences obtained in the present study were deposited in the GenBank database under Accession Numbers KY292417–KY292482.

### Examination of plant growth-promoting traits

To test for isolates with free-living nitrogen-fixing or phosphate-solubilizing activity, fresh colonies grown on nutrient agar were selected and streaked on nitrogen-free agar or tricalcium phosphate agar, respectively. After 5–7 d of cultivation the formation of colonies was recorded. The nitrogen-fixing activities of isolates were assessed as described by Hung *et al*. (24) using an acetylene reduction assay (19). To evaluate the phosphate-solubilizing activities of isolates, colonies were selected and inoculated in tricalcium phosphate medium. After 4 d of cultivation, the supernatant obtained by centrifugation was passed through a 0.45-μm Millipore filter (Merck, Taipei, Taiwan). Soluble phosphate of the cultural filtrate was assessed by the vanadomolybdophosphoric acid colorimetric method (4). To test for isolates with IAA-producing activity, fresh colonies grown on nutrient agar were selected and inoculated in nutrient broth supplemented with 500 μg tryptophan mL^−1^. After cultivation, the production of IAA in the cultural filtrate was assessed by the colorimetric method of Gordon and Weber (15) using pure IAA as the standard.

### Examination of plant constituent-hydrolyzing traits and carbon source utilization patterns

The decomposition of plant constituents was assessed by culturing the substrates tested into nutrient agar, 10-fold diluted nutrient agar, and 100-fold diluted nutrient agar and inoculating the media with isolates. The cellulolytic, pectinolytic, amylolytic, and xylanolytic activities of isolates were evaluated by growing them on media supplemented with 0.5% of carboxymethylcellulose, pectin, soluble starch, and xylan, respectively. Bacterial cultures were cultivated at 30°C for 4 d. The appearance of a clear zone was measured after the addition of specific reagents (Congo red, cetyl trimethyl ammonium bromide, and iodine solution to detect cellulolytic/xylanolytic, pectinolytic, and amylolytic activities, respectively) and then used as an indicator of extracellular enzymatic activities (12, 17, 34, 40). The carbon source utilization patterns of isolates were examined using the GP2/GN2 MicroPlate test panel designed in the Biolog system (Biolog, Hayward, CA, USA) following the manufacturer’s recommendations.

### Assessment of motile and antioxidant traits

Media used for assays of swimming, swarming, and twitching motilities were nutrient broth that contained 0.3, 0.5, and 1% (w/v) of agar, respectively, and motility behaviors were examined as described by Rashid and Kornberg (33). Three oxidants were used to assess the antioxidant traits of bacteria. Each bacterial culture after 24 h of cultivation was spread on nutrient agar, and discs (6 mm in diameter) containing 10 μL of 0.26 M H_2_O_2_, 0.16 M cumene hydroperoxide (CHP), or 0.32 M (tert-butyl hydroperoxide) t-BOOH were placed on nutrient agar plates. After 24 h of cultivation at 30°C, inhibition zones that represent the susceptibility of strains towards different oxidants were measured. The motile and antioxidant traits of strains were tested in triplicate.

### Evaluation of the bacterial inoculation on seedling and mature plant growth

The effects of the bacterial inoculation on seedling growth were assessed in a growth chamber (12-h light/dark cycles) at 25°C for 1 month. The strains tested were grown in 5 mL of nutrient broth for 3 d, and cells were then harvested by centrifugation, washed twice, resuspended in sterile water, and adjusted to an OD_600_ of 1. Seeds were surface sterilized with 30% of H_2_O_2_ for 5 min, rinsed 3 times with sterile water, and soaked at 30°C in sterile water for 12 h. After soaking in the bacterial suspension for 30 min, 3 seeds were placed in each tube containing 10 mL of sterile water agar (0.8% of agar), and another 100 μL of the bacterial suspension was inoculated. After 1 week of cultivation, seedlings were thinned down to one in each tube. After 20 d of cultivation, 100 μL of the bacterial suspension (resuspended in Hoagland solution 1 [22]) was re-inoculated in treatments with the bacterial inoculation. Regarding the un-inoculated treatment represented by Blank (BK), only 100 μL of Hoagland solution 1 was supplemented. Eight replications were conducted for each treatment, and the root and shoot lengths of seedlings were assessed after 1 month of cultivation.

In a pot experiment conducted in a greenhouse, the King Root substrate (N-P_2_O_5_-K_2_O: 1-0.4-0.3, consisting of 92% organic matter, pH 5.0, Dayi Agritech), which was made from raw materials including peat, coconut shell, and lime, was used as medium for plant cultivation. The treatments used were as follows: Control—un-inoculated treatment without chemical fertilization, NPK—un-inoculated treatment with chemical fertilization (1.71 g of KNO_3_, 1.46 g of Ca[H_2_PO_4_]_2_·H_2_O, and 1.34 g of K_2_HPO_4_ kg^−1^ substrate per application), NPK+BF-E14—with chemical fertilization and the strain BF-E14 inoculation, NPK+BF-E16—with chemical fertilization and the strain BF-E16 inoculation, NPK+BF-E18—with chemical fertilization and the strain BF-E18 inoculation, and NPK+BF-E21—with chemical fertilization and the strain BF-E21 inoculation. Three 2-week-germinated seeds were sown at a depth of 2 cm in 1-kg plastic pots (length, 12.6 cm; upper diameter, 14.7 cm; lower diameter, 11.2 cm) filled with substrate. The pots were sprinkled with water. After 1 week of cultivation, seedlings were thinned down to one in each pot, and chemical fertilization was conducted every week until plant harvest. Bacterial cultures suspended in sterile water (*ca*. 10^8^ CFU per inoculation) were inoculated on seeds before sowing as well as on 1-week- and 2-month-grown seedlings in treatments with the bacterial inoculation. Five replications were conducted for each treatment, and plants were watered daily to maintain moisture at field capacity. After 4 months of cultivation, plants were harvested to assess the fresh weights of roots, stems, and leaves. Root samples after drying in an oven at 70°C for 72 h were further freeze-dried and sent for polyphenol and antioxidant activity (superoxide dismutase [SOD], α,α-diphenyl-β-picrylhydrazyl [DPPH], and Trolox equivalent anti-oxidant capacity [TEAC]) analyses, which were conducted by the Bioresource Collection and Research Center, Food Industry Research and Development Institute, Taiwan.

The results obtained for seedling and mature plant growth were presented as mean values, and a one-way ANOVA (analysis of variance) and Duncan’s test (*P*<0.05) were used to evaluate the significance of differences between treatments using XLSTAT Statistical Software (New York, NY, USA).

## Results and Discussion

### Phylogenetic analysis of isolates obtained from the rhizosphere or root interior

Roots, the part of the plant primarily responsible for nutrient uptake, provide specific niches for bacteria. The rhizospheric effects that enrich root-associated bacteria play important roles in ecological functions, such as nutrient transformation and organic matter decomposition. Investigations on these bacteria provide insights into their interactions with hosts and contribute to the development of suitable bioinoculants used in plant cultivation. In the present study, 42 and 24 isolates were obtained from the rhizosphere and root interior, respectively (Tables 1, S1, and S2). Few bacterial lineages were found in all three cultural media, and most were only enriched and obtained on a single culture medium. This result demonstrated the usefulness of multiple media to obtain a broader range of bacterial lineages. Isolates inhabiting the rhizosphere of *P. grandiflorum* were affiliated with the genera *Bacillus*, *Burkholderia*, *Cellulosimicrobium*, *Chromobacterium*, *Citrobacter*, *Leifsonia*, *Microbacterium*, *Mitsuaria*, *Novosphingobium*, *Paenarthrobacter*, *Paraburkholderia*, *Pseudomonas*, *Rhizobium*, and *Sphingopyxis*. Regardless of the culture medium used for isolation, members belonging to the phylum *Proteobacteria* were the dominant bacterial group (8 out of 14 isolates obtained on nutrient agar, 12 out of 17 isolates obtained on nitrogen-free agar, and all isolates obtained on tricalcium phosphate agar) (Table 1 and Fig. S1). The root interior harbored members affiliated with the genera *Bacillus*, *Burkholderia*, *Klebsiella*, *Microbacterium*, *Pantoea*, *Rhizobium*, and *Xenophilus*. Most of these species belonged to the phylum *Proteobacteria* (22 out of 24 isolates), which still represented the dominant bacterial group isolated from the root interior (Fig. S2). The present study demonstrated that the roots of *P. grandiflorum* were inhabited by various bacterial populations (more than 30 species), which have not been reported previously, particularly for non-negligible *Proteobacteria*. *Proteobacteria* has also been identified as the most dominant phylum in the rhizosphere and root interior from various plants (21, 28). We also found that the most prevalent strains associated with *P. grandiflorum* roots belonged to the genus *Rhizobium* (17 out of 66 isolates), which has been extensively studied as representative plant-associated microbes (43). More than 10 isolates obtained here shared less than 99% sequence similarities in 16S rDNA with their most closely related species. They may be novel species that need to be examined in more detail in the future.

A number of species belonging to *Actinobacteria* and *α-Proteobacteria* were obtained on nitrogen-free agar in the present study, demonstrating their potential to fix atmospheric nitrogen. They may also survive in nitrogen-limited medium, which may be related to their potentially facultatively oligotrophic nature (23). In consideration of practical applications, only six strains, namely *Microbacterium* sp. BF-E14, *Rhizobium* sp. BF-E15, *Rhizobium* sp. BF-E16, *Rhizobium azooxidifex* BF-E17, *Rhizobium* sp. BF-E18, and *Xenophilus azovorans* BF-E21, which were isolated from the root interior and with fewer biosafety concerns, were selected and examined in subsequent experiments. Five out of the 6 strains showed facultatively oligotrophic behavior because they formed colonies on both nutrient-rich and nutrient-poor media (Table 2). Among these, strain BF-E17, which was closely related to *R*. *azooxidifex*, showed markedly better proliferation on 1,000-fold diluted nutrient agar than the other strains examined.

### Plant growth-promoting traits of selected strains

Six endophytic strains were subjected to plant growth-promoting characterization in order to evaluate their potential to promote plant growth. All of these strains grew on nitrogen-free agar and tricalcium phosphate agar (Table 2). Five out of the 6 strains changed the color of medium from green to blue, which may function as an indicator of ammonia production in nitrogen-free agar. The acetylene reduction assay confirmed that these strains exhibited free-living nitrogen-fixing activities, ranging between 1.2 and 2.34 nmol ethylene tube^−1^ h^−1^. Five out of the 6 strains produced 6.5 to 859.1 μg mL^−1^ soluble phosphate in culture medium after 4 d of cultivation, while strain BF-E21, which was closely related to *X. azovorans*, failed to solubilize tricalcium phosphate in the liquid medium tested. All of the tested strains produced 17.6 to 56.9 μg mL^−1^ IAA in tryptophan-containing nutrient broth after 4 d of cultivation. Many plant growth-promoting traits are commonly used as indicators to screen bacteria with plant growth-promoting potential. However, a correlation has yet to be confirmed between the possession of these traits and plant growth-promoting/inhibiting effects, which needs to be established through co-cultivation experiments to identity PGPB from plant growth-inhibiting bacteria (PGIB) (25, 35).

### Plant constituent-hydrolyzing traits and carbon source utilization patterns of selected strains

To elucidate the mechanisms underlying the entrance and survival of these root-inhabited bacteria via plant constituent hydrolysis, several plant polymers were separately used as substrates in screening agar plates. Strain BF-E14 hydrolyzed cellulose, pectin, starch, and xylan, which was confirmed by the appearance of a clear zone around colonies on each screening agar plate (Table 3). All of the *Rhizobium* strains tested (BF-E15, BF-E16, BF-E17, and BF-E18) also utilized cellulose, but not xylan. Among these, strains BF-E15 and BF-E18 hydrolyzed pectin, while strain BF-E16 utilized starch. Regarding strain BF-E21, only pectin was utilized. The penetration of endophytic bacteria into the host plant was mediated by hydrolytic enzymes, such as cellulase and pectinase (18). However, the constitutive release of plant cell wall-degrading enzymes by endophytic bacteria is undesirable because it confers plant pathogenicity (7). Verma and his colleagues (42) demonstrated that a strain with low cellulase and pectinase, but high nitrogen-fixing activities may be a useful endophytic diazotroph for studies on the colonization of rice varieties. In consideration of carbon source utilization patterns assessed by the Biolog system, 14 carbon sources were utilized well by strain BF-E14 (Table 3). Dissimilar carbon sources that may be categorized as carboxylic acid and amino acids were weakly utilized by strain BF-E21, while strain BF-E14 preferred carbohydrate as its carbon source. No well-utilized carbon sources were recorded in any of the rhizobial strains tested after 2 d of cultivation, while a few carbon sources were weakly utilized by some strains.

### Motile and antioxidant traits of selected strains

**Fig. 1:**
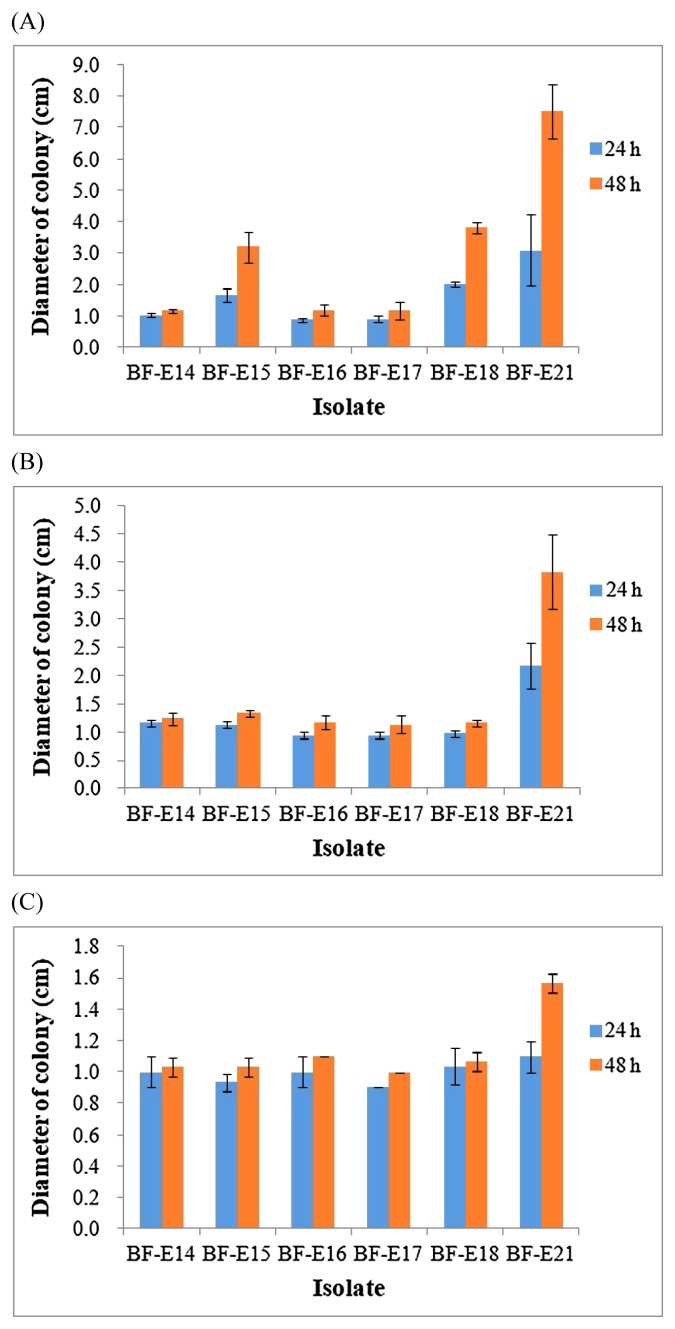
Motile behavior of 6 selected strains on (A) swim plates, (B) swarm plates, and (C) twitch plates within 2 d of cultivation. Error bars show the standard deviation, *n*=3.

An abundant amount of experimental evidence has shown that chemotaxis enhances the ability of soil bacteria to colonize the roots of diverse plant hosts (37). Endophytic bacteria have developed many ROS-scavenging enzymes under biotic or abiotic stress conditions (1, 28). Motile and antioxidant traits may contribute to niche adaptation for bacteria to survive in plants, which may further augment the influence of bacteria on whole plant growth. In the present study, swimming motility was recorded for strains BF-E15 and BF-E18 (Fig. 1). Strain BF-E21 showed not only swimming, but also swarming and twitching motilities. No apparent motile behavior was found in the 3 other strains. Regarding antioxidant traits, strain BF-E14 was susceptible to H_2_O_2_, but resistant to CHP and t-BOOH (Table 4). Strain BF-E15 was susceptible to H_2_O_2_ and CHP, but resistant to t-BOOH, while strain BF-E18 showed the opposite antioxidant pattern. Strains BF-E16 and BF-E17, which were non-motile under the conditions tested, both showed resistance to H_2_O_2_, CHP, and t-BOOH. Strain BF-E21 was susceptible to all of the oxidants tested, but may evade oxidative stress through its motile behavior.

### Effects of the bacterial inoculation on seedling and mature plant growth

**Fig. 2:**
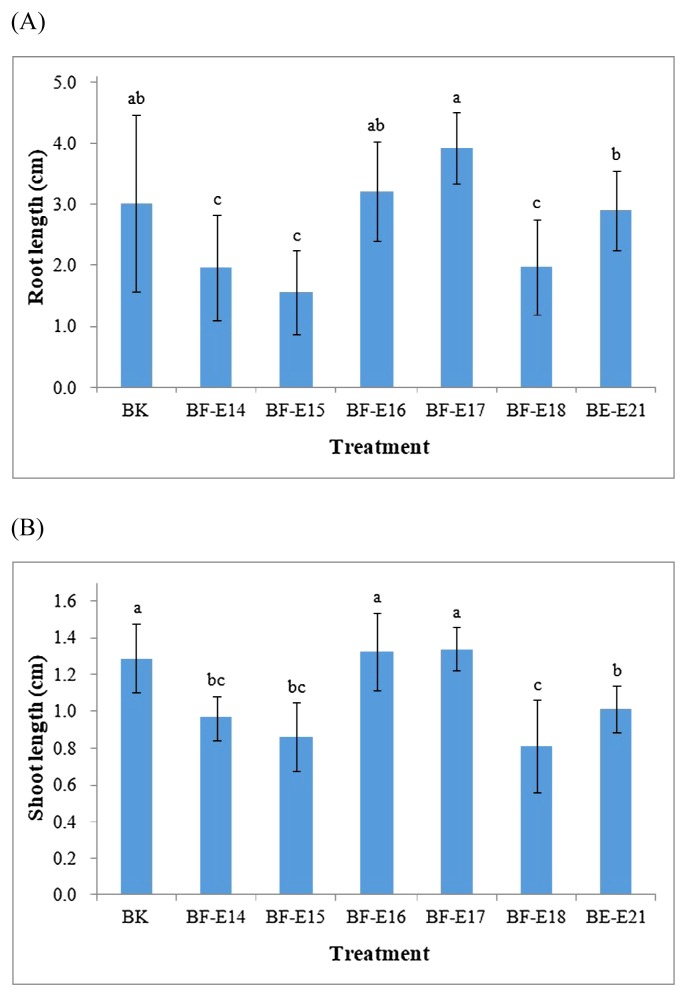
Effects of the bacterial inoculation on the (A) root length and (B) shoot length of *Platycodon grandiflorum* after 1 month of cultivation. Blank (BK)—un-inoculated treatment. Error bars showed the standard deviation, *n*=8. Lengths with the same letters above bars were not significantly different.

**Fig. 3:**
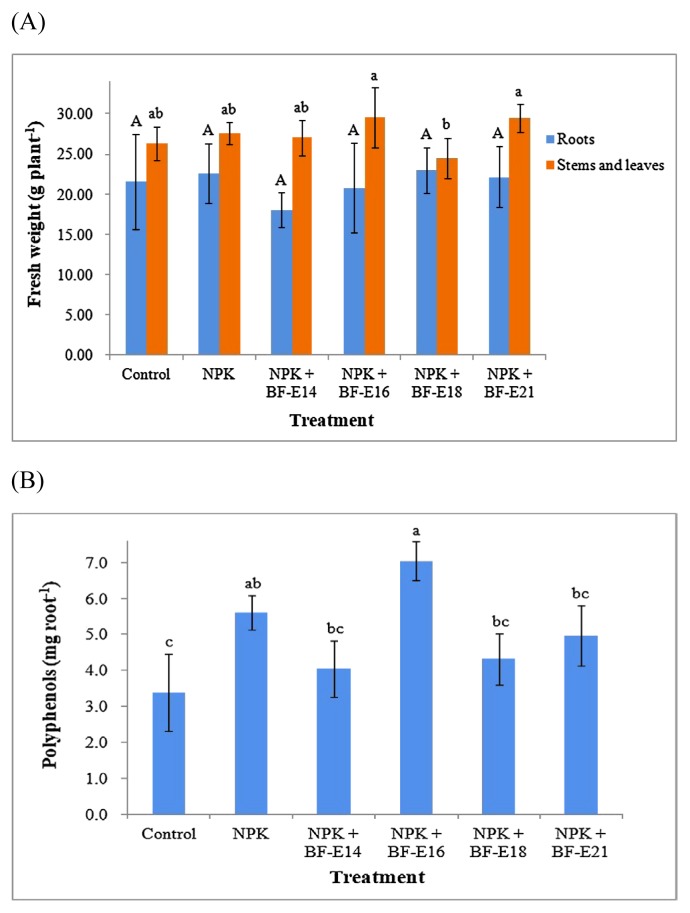
Effects of various treatments on the (A) fresh weight and (B) polyphenol content of *Platycodon grandiflorum* after 4 months of cultivation. Control—un-inoculated treatment without chemical fertilization, NPK—un-inoculated treatment with chemical fertilization. Error bars show the standard deviation, *n*=5. Lengths with the same letters above bars were not significantly different.

Six endophytic strains were inoculated separately on the seeds of *P. grandiflorum*, and seedling growth was assessed after 1 month of cultivation. Strains BF-16 and BF-E17, which did not exhibit pectinolytic activities, showed similar root and shoot lengths for seedlings to the un-inoculated (BK) treatment, demonstrating their compatibility with the host plant (Fig. 2). The inoculation with strain BF-E21 decreased the shoot length of seedlings. The other 3 treatments, namely, inoculations with strains BF-E14, BF-E15, and BF-E18, were all detrimental to the root and shoot growth of seedlings, and this may be related to their abilities to hydrolyze both cellulose and pectin. Regarding strains exerting various effects (positive or negative) on seedling growth, 4 strains (BF-E14, BF-E16, BF-E18, and BF-E21) with different plant constituent-hydrolyzing patterns were selected and used in a 4-month pot experiment. The performance of the bacterial inoculation along with chemical fertilization on the growth of *P. grandiflorum* was evaluated. The BF-E16 or BF-E21 inoculation resulted in a slightly higher aboveground biomass than the un-inoculated treatment with NPK fertilization (Fig. 3). The inoculation with strain BF-E14 or BF-E16, which hydrolyzed root storage starch, decreased the fresh weight of roots, while the fresh weights of stems and leaves were the lowest with the NPK+BF-E18 treatment. The hydrolysis of plant constituents by these strains may be one of the factors contributing to the negative effects observed on plant growth. These strains, which suppress plant growth without inducing any apparent pathogenic symptoms, may be regarded as plant growth-inhibiting bacteria (25). Since hydrolytic enzymes are also produced by plant pathogens, further information on their regulation and expression is needed to distinguish endophytic bacteria from plant pathogens (42).

In the present study, the BF-E16 inoculation resulted in a slightly higher root polyphenol content than the un-inoculated treatment with NPK fertilization (Fig. 3). The BF-E16 inoculation also slightly increased antioxidant activities (SOD, DPPH, and TEAC), which may contribute to the greater radical-scavenging potential of *P. grandiflorum* (Table 5). The present results also demonstrated that strain BF-E16 was resistant to all of the oxidants tested in cultural media under oxidative stress. Although the up-regulation of antioxidants may inhibit the growth of plants through cross-talk between developmental and stress-response networks (5), the NPK+BF-E16 treatment resulted in similar plant growth to the NPK treatment.

Although *Rhizobium* species, such as *R. oryzae*, *R. oryzicola*, and *R. oryziradicis*, were identified as endophytes in rice, their influence on the growth of non-leguminous plants remains unclear (31, 46, 47). In the present study, 4 *Rhizobium* strains (BF-E15, BF-E16, BF-E17, and BF-E18) from different phylogenetic groups (Fig. S2) were selected and compared, which provided evidence to show that different *Rhizobium* species may possess distinct traits for plant growth promotion and niche adaptation. These traits also contributed to the various effects observed on the seedling and mature plant growth of the non-leguminous medicinal plant *P. grandiflorum*. Among all of the strains tested, the potentially novel *Rhizobium* sp. BF-E16 (sharing 98.35% sequence similarity in 16S rDNA with its most closely related species *R. paranaense*) was identified as a compatible strain to cultivate *P. grandiflorum*.

In conclusion, diverse bacterial lineages that have not been reported as *P. grandiflorum*-associated bacteria were examined in the present study, particularly members belonging to the non-negligible phylum *Proteobacteria*. The selected strains that exhibited nitrogen-fixing or phosphate-solubilizing and IAA-producing activities were beneficial or detrimental to plant growth, as evidenced by their effects on the length of seedlings and biomass of mature plants. Among 4 endophytic *Rhizobium* species, the potentially novel *Rhizobium* sp. BF-E16, which was more compatible with the non-leguminous medicinal plant *P. grandiflorum*, was identified. In addition to plant growth-promoting traits, characteristics such as plant constituent-hydrolyzing activities need to be taken into consideration and their roles clarified when investigating plant growth-promoting rhizobacteria.

## SUPPLEMENTARY MATERIAL



## Figures and Tables

**Table 1:** Affiliation of 66 isolates obtained on various cultural media from the rhizosphere or root interior of *Platycodon grandiflorum*.

Bacterial lineage	Isolation from the rhizosphere			Isolation from the root interior		
	
	Nutrient agar	Nitrogen-free agar	Tricalcium phosphate agar	Nutrient agar	Nitrogen-free agar	Tricalcium phosphate agar
***Actinobacteria; Actinobacteria* (Phylum; Class)**	2/14^a^	3/17	0/11	1/8	0/9	0/7
*Cellulosimicrobium* sp.		1				
*Leifsonia shinshuensis*		1				
*Microbacterium* sp.	2			1		
*Paenarthrobacter nitroguajacolicus*		1				
***Firmicutes; Bacilli***	4/14	2/17	0/11	1/8	0/9	0/7
*Bacillus aryabhattai*		2				
*Bacillus bataviensis*	1					
*Bacillus cereus*				1		
*Bacillus* sp.	3					
***Proteobacteria; α-Proteobacteria***	2/14	8/17	0/11	3/8	3/9	3/7
*Novosphingobium rhizosphaerae*		1				
*Rhizobium azooxidifex*				1		
*Rhizobium cauense*		1				
*Rhizobium daejeonense*		1				
*Rhizobium miluonense*		1				
*Rhizobium radiobacter*		1				
*Rhizobium rhizoryzae*		1				
*Rhizobium tropici*		1				
*Rhizobium* sp.	1	1		2	3	3
*Sphingopyxis flava*	1					
***Proteobacteria; β-Proteobacteria***	4/14	3/17	8/11	2/8	3/9	1/7
*Burkholderia cenocepacia*	2		8	2	2	1
*Burkholderia* sp.		2				
*Chromobacterium violaceum*	1					
*Mitsuaria chitosanitabida*		1				
*Paraburkholderia sacchari*	1					
*Xenophilus azovorans*					1	
***Proteobacteria; γ-Proteobacteria***	2/14	1/17	3/11	1/8	3/9	3/7
*Citrobacter bitternis*			1			
*Klebsiella pneumoniae*					1	
*Pantoea dispersa*				1	2	3
*Pseudomonas aeruginosa*	1	1	1			
*Pseudomonas nitroreducens*			1			
*Pseudomonas* sp.	1					

a Number of isolates within the representative class out of all isolates obtained from the same culture medium.

**Table 2:** Culture and plant growth-promoting traits of 6 selected strains.

Strain	Facultative oligotrophy		Nitrogen fixation		Phosphate solubilization		Production of IAA in tryptophan-containing nutrient broth (μg mL^−1^)
		
	Growth on nutrient agar	Growth on 1,000-fold diluted nutrient agar	Growth on nitrogen-free agar/color of medium	Nitrogen-fixing activity (nmol ethylene tube^−1^ h^−1^)	Growth on tricalcium phosphate agar	Phosphate- solubilizing activity (μg mL^−1^)	
BF-E14	+	(+)	+/Blue	2.02	+	72.0	56.9
BF-E15	+	(+)	+/Blue	1.89	+	115.3	17.6
BF-E16	+	(+)	+/Blue	2.34	+	6.7	21.6
BF-E17	+	+	+/Green	0.06	+	6.5	19.6
BF-E18	+	(+)	+/Blue	1.93	+	859.1	21.6
BF-E21	+	−	+/Blue	1.20	+	0	54.9

+; formation of apparent colonies, (+); formation of small and non-apparent colonies, −; no growth.

**Table 3:** Plant constituent-hydrolyzing and carbon source-utilizing traits of 6 selected strains.

Strain	Plant constituent hydrolysis				Carbon source utilization	

	Cellulose	Pectin	Starch	Xylan	Well-utilized carbon source (OD>0.5)	Weakly utilized carbon source (0.35<OD<0.5)
BF-E14	+^a^	+	+	+	N-Acetyl-D-Glucosamine, Arbutin, D-Cellobiose, D-Fructose, α-D-Glucose, Maltotriose, D-Mannitol, D-Mannose, Sucrose, D-Trehalose, Turanose, L-Alaninamide, Adenosine, Inosine	Tween 40, L-Alanine
BF-E15	+	+	−	−	ND^b^	D-Fructose, α-D-Glucose, Formic Acid
BF-E16	+	−	+	−	ND	ND
BF-E17	+	−	−	−	ND	Maltose, D-Mannitol
BF-E18	+	+	−	−	ND	ND
BF-E21	−	+	−	−	ND	β-Hydroxybutyric Acid, D, L-Lactic Acid, Propionic Acid, L-Aspartic Acid, L-Glutamic Acid

a +; positive for a clear zone around the colony, −; negative for a clear zone around the colony.

b ND; not detectable on the Biolog GP/GN microplate.

**Table 4:** Antioxidant traits of 6 selected strains.

Strain	0.26 M H_2_O_2_	0.16 M CHP	0.32 M t-BOOH
BF-E14	Susceptible	Resistant	Resistant
BF-E15	Susceptible	Susceptible	Resistant
BF-E16	Resistant	Resistant	Resistant
BF-E17	Resistant	Resistant	Resistant
BF-E18	Resistant	Resistant	Susceptible
BF-E21	Susceptible	Susceptible	Susceptible

Susceptible; positive for a clear zone around oxidant-containing discs, resistant; negative for a clear zone around oxidant-containing discs.

**Table 5:** Effects of various treatments on antioxidant activities of *Platycodon grandiflorum* roots after 4 months of cultivation.

Treatment	SOD (%)	DPPH (%)	TEAC (%)/Trolox (mM)
Control^a^	29.6±2.7 ab^b^	29.1±1.1 a	8.4±1.3 c/0.14±0.11 a
NPK	30.8±4.5 ab	27.6±0.4 ab	10.4±1.2 bc/0.18±0.12 a
NPK+BF-E14	31.7±3.4 ab	27.6±0.9 ab	13.1±1.1 ab/0.23±0.06 a
NPK+BF-E16	36.2±5.1 a	29.2±3.6 a	14.9±1.2 a/0.27±0.12 a
NPK+BF-E18	29.4±1.6 ab	26.4±1.9 ab	12.2±1.8 b/0.22±0.08 a
NPK+BF-E21	27.1±3.3 b	25.3±2.0 b	11.5±1.8 b/0.20±0.08 a

a Control—un-inoculated treatment without chemical fertilization, NPK—un-inoculated treatment with chemical fertilization.

b In columns, means followed by the same letters were not significantly different.
