# Supplementary material for: Exploration of Root-associated Bacteria from the Medicinal Plant Platycodon grandiflorum
Source: Microbes Environ. 2019 Dec 27;34(4):413–20. doi: 10.1264/jsme2.ME19030 (PMC6934386; doi:10.1264/jsme2.ME19030)
Supplement: Supplementary file 1 [file 34_413_s1.pdf]

Supplementary data

Legend to Supplementary Figures

Fig. S1. Neighbor-joining tree with bootstrapping 1000 replicates showing the phylogenetic relationship of 42 isolates obtained from rhizosphere of *Platycodon grandiflorum* and their most closely-related species based on 16S rDNA sequences.

Fig. S2. Neighbor-joining tree with bootstrapping 1000 replicates showing the phylogenetic relationship of 24 isolates obtained from root interior of *Platycodon grandiflorum* and their most closely-related species based on 16S rDNA sequences.

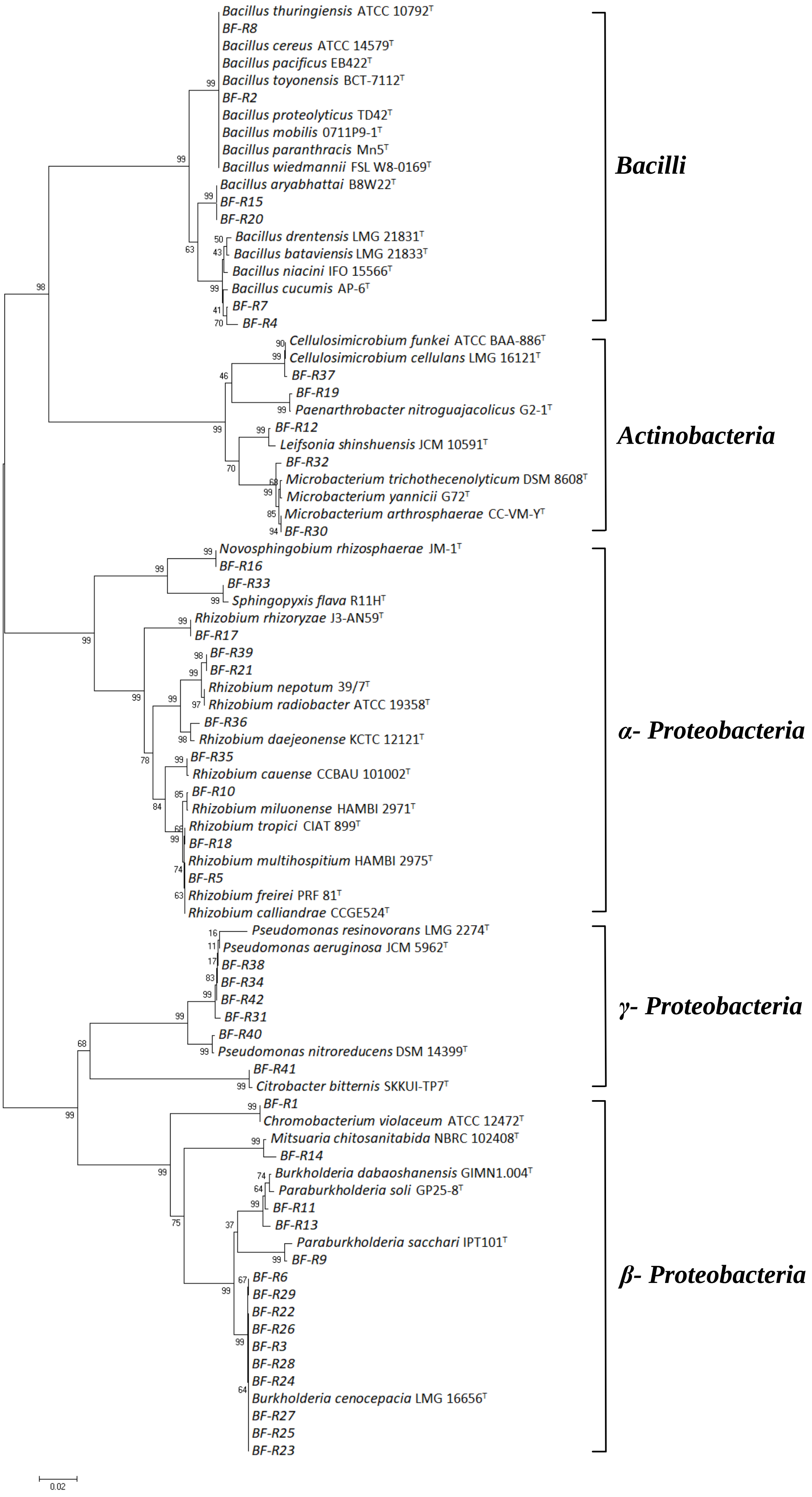

Fig. S1 (Huang et al.)

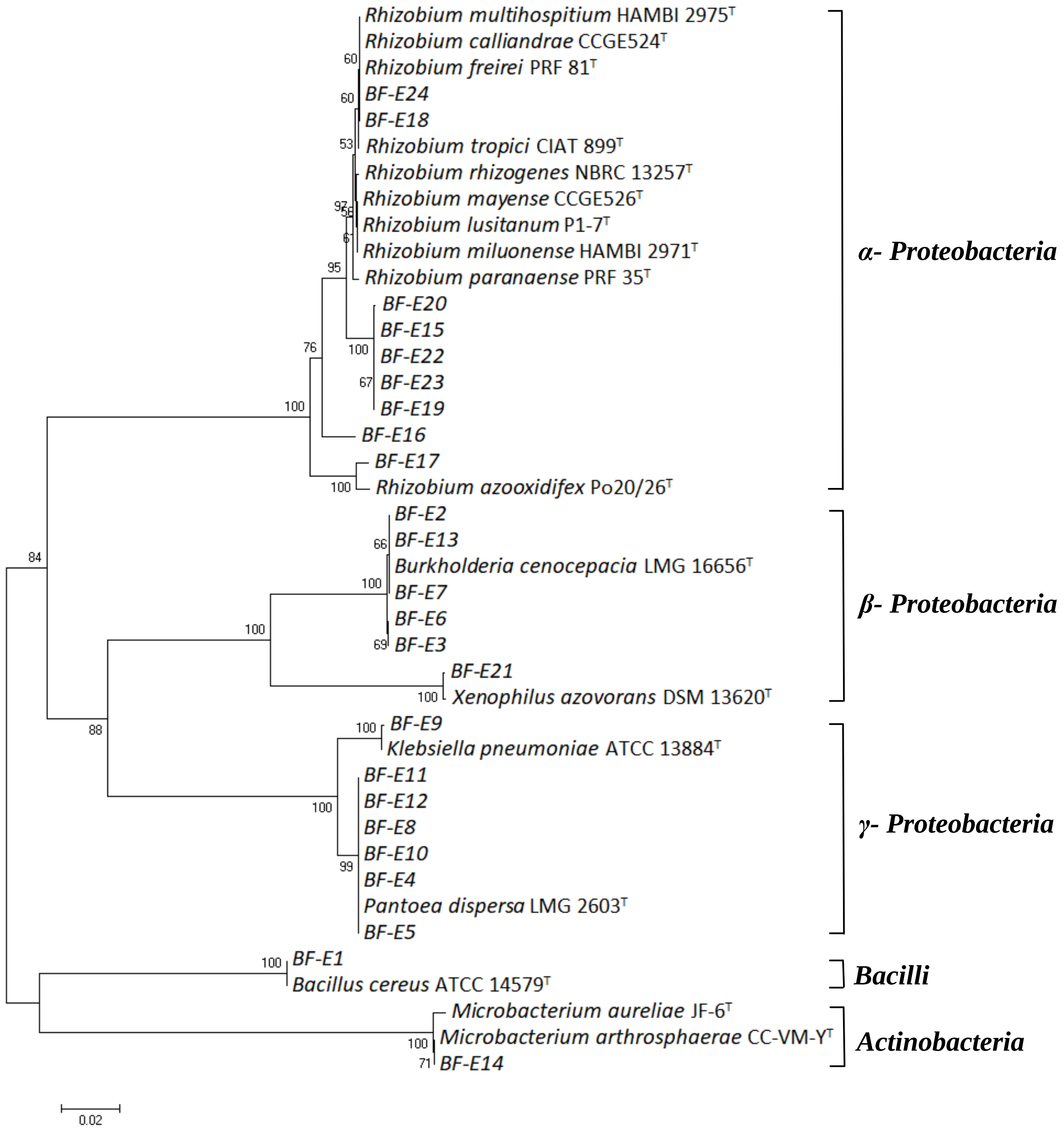

Fig. S2 (Huang et al.)

Table S1. Designation and identification of 42 isolates obtained from rhizosphere of *Platycodon grandiflorum*.

Strain name	Top-hit taxon and strain	Similarity (%)	Diff/Total nt	Medium used for isolation
**<u>Pot A</u>**				
BF-R1	*Chromobacterium violaceum* ATCC 12472^T	99.93	1/1458	
BF-R2	*Bacillus cereus* ATCC 14579^T	100	0/1054	
	Bacillus mobilis 0711P9-1^T			
	Bacillus pacificus EB422^T			
	Bacillus paranthracis Mn5^T			
	Bacillus proteolyticus TD42^T			
	Bacillus thuringiensis ATCC 10792^T			
	Bacillus toyonensis BCT-7112^T			
	Bacillus wiedmannii FSL W8-0169^T			
BF-R3	*Burkholderia cenocepacia* LMG 16656^T	100	0/1042	
BF-R4	*Bacillus bataviensis* LMG 21833^T	98.60	20/1430	
BF-R5	*Rhizobium calliandrae* CCGE524^T	100	0/1055	Nutrient agar
	Rhizobium freirei PRF 81^T			
	Rhizobium multihospitium HAMBI 2975^T			
BF-R6	*Burkholderia cenocepacia* LMG 16656^T	99.90	1/1048	
BF-R7	*Bacillus cucumis* AP-6^T	99.04	14/1451	
	Bacillus drentensis LMG 21831^T	99.01	14/1408	
	Bacillus bataviensis LMG 21833^T	98.91	16/1472	
	Bacillus niacini IFO 15566^T			
BF-R8	*Bacillus cereus* ATCC 14579^T	100	0/1051	
	Bacillus mobilis 0711P9-1^T			
	Bacillus pacificus EB422^T			
	Bacillus paranthracis Mn5^T			

	Bacillus proteolyticus TD42^{T}			
	Bacillus thuringiensis ATCC 10792^{T}			
	Bacillus toyonensis BCT-7112^{T}			
	Bacillus wiedmannii FSL W8-0169^{T}			
BF-R9	*Paraburkholderia sacchari* IPT101^{T}	99.31	10/1455	
BF-R10	*Rhizobium miluonense* HAMBI 2971^{T}	99.93	1/1403	
BF-R11	*Burkholderia dabaoshanensis* GIMN1.004^{T}	98.81	17/1430	
	Paraburkholderia soli GP25-8^{T}	98.62	20/1453	
BF-R12	*Leifsonia shinshuensis* JCM 10591^{T}	99.51	7/1430	
BF-R13	*Burkholderia dabaoshanensis* GIMN1.004^{T}	98.75	13/1040	
	Paraburkholderia soli GP25-8^{T}			
BF-R14	*Mitsuaria chitosanitabida* NBRC 102408^{T}	99.38	9/1451	Nitrogen-free agar
BF-R15	*Bacillus aryabhattai* B8W22^{T}	100	0/1474	
BF-R16	*Novosphingobium rhizosphaerae* JM-1^{T}	100	0/1382	
BF-R17	*Rhizobium rhizoryzae* J3-AN59^{T}	100	0/1403	
BF-R18	*Rhizobium tropici* CIAT 899^{T}	99.71	4/1403	
BF-R19	*Paenarthrobacter nitroguajacolicus* G2-1^{T}	99.65	5/1442	
BF-R20	*Bacillus aryabhattai* B8W22^{T}	99.90	1/1048	
BF-R21	*Rhizobium radiobacter* ATCC 19358^{T}	99.72	4/1407	
BF-R22	*Burkholderia cenocepacia* LMG 16656^{T}	100	0/1043	
BF-R23	*Burkholderia cenocepacia* LMG 16656^{T}	100	0/1046	Tricalcium phosphate agar
BF-R24	*Burkholderia cenocepacia* LMG 16656^{T}	100	0/1046	
BF-R25	*Burkholderia cenocepacia* LMG 16656^{T}	100	0/1042	

BF-R26	*Burkholderia cenocepacia* LMG 16656^T	100	0/1043	
BF-R27	*Burkholderia cenocepacia* LMG 16656^T	100	0/1045	
BF-R28	*Burkholderia cenocepacia* LMG 16656^T	100	0/1042	
BF-R29	*Burkholderia cenocepacia* LMG 16656^T	99.90	1/1047	
**<u>Pot B</u>**				
BF-R30	*Microbacterium arthrosphaerae* CC-VM-Y^T	100	0/1005	
	Microbacterium trichothecenolyticum DSM 8608^T	99.71	3/1052	
	Microbacterium yannicii G72^T			
BF-R31	*Pseudomonas resinovorans* LMG 2274^T	97.93	30/1450	
	Pseudomonas aeruginosa JCM 5962^T	97.81	32/1458	Nutrient agar
BF-R32	*Microbacterium arthrosphaerae* CC-VM-Y^T	99.50	5/1005	
	Microbacterium trichothecenolyticum DSM 8608^T	99.33	7/1049	
	Microbacterium yannicii G72^T			
BF-R33	*Sphingopyxis flava* R11H^T	99.72	4/1411	
BF-R34	*Pseudomonas aeruginosa* JCM 5962^T	100	0/1047	
BF-R35	*Rhizobium cauense* CCBAU 101002^T	99.93	1/1403	
BF-R36	*Rhizobium daejeonense* KCTC 12121^T	99.43	8/1407	
BF-R37	*Cellulosimicrobium funkei* ATCC BAA-886^T	99.79	3/1443	Nitrogen-free agar
	Cellulosimicrobium cellulans LMG 16121^T	99.72	4/1444	
BF-R38	*Pseudomonas aeruginosa* JCM 5962^T	100	0/1042	

BF-R39	*Rhizobium nepotum* 39/7^T<br>*Rhizobium radiobacter* ATCC 19358^T	99.62	4/1047	
BF-R40	*Pseudomonas nitroreducens* DSM 14399^T	99.45	8/1457	
BF-R41	*Citrobacter bitternis* SKKUI-TP7^T	99.63	5/1358	Tricalcium phosphate agar
BF-R42	*Pseudomonas aeruginosa* JCM 5962^T	100	0/1044	

Table S2. Designation and identification of 24 isolates obtained from root interior of *Platycodon grandiflorum*.

Strain name	Top-hit taxon and strain	Similarity (%)	Diff/Total nt	Medium used for isolation
**<u>Pot A</u>**				
BF-E1	*Bacillus cereus* ATCC 14579^T	100	0/1474	Nutrient agar
BF-E2	*Burkholderia cenocepacia* LMG 16656^T	99.93	1/1455	
BF-E3	*Burkholderia cenocepacia* LMG 16656^T	99.90	1/1045	
BF-E4	*Pantoea dispersa* LMG 2603^T	100	0/1041	
BF-E5	*Pantoea dispersa* LMG 2603^T	100	0/1343	Nitrogen-free agar
BF-E6	*Burkholderia cenocepacia* LMG 16656^T	99.90	1/1048	
BF-E7	*Burkholderia cenocepacia* LMG 16656^T	100	0/1045	
BF-E8	*Pantoea dispersa* LMG 2603^T	100	0/1042	
BF-E9	*Klebsiella pneumoniae* subsp. *rhinoscleromatis* ATCC 13884^T	99.66	5/1460	
BF-E10	*Pantoea dispersa* LMG 2603^T	100	0/1041	Tricalcium phosphate agar
BF-E11	*Pantoea dispersa* LMG 2603^T	100	0/1040	
BF-E12	*Pantoea dispersa* LMG 2603^T	100	0/1039	
BF-E13	*Burkholderia cenocepacia* LMG 16656^T	100	0/1039	
**<u>Pot B</u>**				
BF-E14	*Microbacterium arthrosphaerae* CC-VM-Y^T	100	0/1339	Nutrient agar
	Microbacterium aureliae JF-6^T	98.96	15/1445	
BF-E15	*Rhizobium freirei* PRF 81^T	98.93	15/1403	
	Rhizobium miluonense HAMBI 2971^T			
	Rhizobium multihospitium HAMBI 2975^T			
BF-E16	*Rhizobium paranaense* PRF 35^T	98.35	23/1392	
	Rhizobium rhizogenes NBRC 13257^T	98.15	26/1403	
BF-E17	*Rhizobium azooxidifex* Po20/26^T	99.06	13/1390	
BF-E18	*Rhizobium freirei* PRF 81^T	100	0/1403	Nitrogen-free

	Rhizobium multihospitium HAMBI 2975^{T}			agar
BF-E19	*Rhizobium calliandrae* CCGE524^{T}<br>*Rhizobium freirei* PRF 81^{T}<br>*Rhizobium lusitanum* P1-7^{T}<br>*Rhizobium mayense* CCGE526^{T}<br>*Rhizobium miluonense* HAMBI 2971^{T}<br>*Rhizobium multihospitium* HAMBI 2975^{T}	98.57	15/1049	
BF-E20	*Rhizobium calliandrae* CCGE524^{T}<br>*Rhizobium freirei* PRF 81^{T}<br>*Rhizobium lusitanum* P1-7^{T}<br>*Rhizobium mayense* CCGE526^{T}<br>*Rhizobium miluonense* HAMBI 2971^{T}<br>*Rhizobium multihospitium* HAMBI 2975^{T}<br>*Rhizobium rhizogenes* NBRC 13257^{T}<br>*Rhizobium tropici* CIAT 899^{T}	98.47	16/1048	
BF-E21	*Xenophilus azovorans* DSM 13620^{T}	99.86	2/1455	
BF-E22	*Rhizobium calliandrae* CCGE524^{T}<br>*Rhizobium freirei* PRF 81^{T}<br>*Rhizobium lusitanum* P1-7^{T}<br>*Rhizobium mayense* CCGE526^{T}<br>*Rhizobium miluonense* HAMBI 2971^{T}<br>*Rhizobium multihospitium* HAMBI 2975^{T}	98.57	15/1047	Tricalcium phosphate agar
BF-E23	*Rhizobium calliandrae* CCGE524^{T}<br>*Rhizobium freirei* PRF 81^{T}<br>*Rhizobium lusitanum* P1-7^{T}	98.57	15/1050	

	Rhizobium mayense CCGE526^T		
	Rhizobium miluonense HAMBI 2971^T		
	Rhizobium multihospitium HAMBI 2975^T		
BF-E24	*Rhizobium calliandrae* CCGE524^T	100	0/1047
	Rhizobium freirei PRF 81^T		
	Rhizobium multihospitium HAMBI 2975^T		
